# “An antibiotic turned contraceptive”: The tale of ampicillin‐cloxacillin

**DOI:** 10.1002/hsr2.481

**Published:** 2022-01-13

**Authors:** Chinonyerem O. Iheanacho

**Affiliations:** ^1^ Department of Clinical Pharmacy and Public Health, Faculty of Pharmacy University of Calabar Calabar Nigeria

**Keywords:** ampicillin‐cloxacillin, antibiotics, contraception, emergency contraceptive, misuse, Nigeria

## Abstract

Antibiotic misuse is a major public health threat globally, requiring active efforts toward halting its progression. The African sub‐region is worst hit by communicable diseases, and also possesses weak health systems to effectively combat the menace of antimicrobial resistance. Meanwhile, antibiotics are among the most regularly misused medicines in Nigeria, particularly for inappropriate indication and duration. This is seen in its use as a contraceptive by young women in the country. This paper evaluates the reliance and use of an antibiotic for contraception, a practice that has continuously being a large source of antibiotic misuse in Nigeria. Its causes and effects are also evaluated in this commentary. To effectively combat the increasing antimicrobial misuse, correct use of antibiotics for their appropriate indications is highly essential. Adequate knowledge of contraception should be ensured among young women in developing countries, particularly in Nigeria.

## 1 INTRODUCTION

There is crucial and urgent need to halt the ongoing widespread reliance and use of ampicillin‐cloxacillin (Ampiclox®) as an emergency contraceptive among young women in Nigeria. One capsule of branded ampicillin‐cloxacillin taken after unprotected sex is misconstrued to be effective against conception.^1^, ^2^, ^3^ This unproven folkloric practice cuts across young women of various geographical location in the country,^1^, ^2^, ^3^ and poses unprecedented risk to the rapid increase in the global menace of antimicrobial resistance. Ampicillin‐cloxacillin is an antibiotic proven to be effective against gram positive and some gram negative microorganisms. It is specifically active for the prophylaxis and treatment of several bacterial diseases caused by susceptible microorganisms, and no scientific data or information has proven its effectiveness against conception. Meanwhile, several approved contraceptive methods are available, but appear to be neglected or underutilized. Over‐the‐counter availability of emergency contraceptives has also not halted their replacement with this antibiotic. It is imperative to note that the poor adoption of modern contraceptive methods by young women and their partners in Nigeria, and the repurposing of an antibiotic for contraception calls for public health concerns and immediate attention from stakeholders.

## 2 CAUSES OF THIS ANTIBIOTIC MISUSE

“Chaotic drug distribution” in Nigeria^4^ which has promoted misuse of several drugs^5^ is a leading cause of this misuse. Poor commitment to antimicrobial stewardship,^6^ non‐regulation, ease of access, and over‐the‐counter availability of antibiotics are also major promoters of this practice in the country. Low awareness of effective and modern contraceptive methods also accounts for this trend of antibiotic misuse.^2^ Cultural barriers to sexuality education and low access to reproductive health education, particularly in rural and sub‐urban regions of the country are also important contributors to this highly prevalent antibiotic misuse. Notable cultural barriers hindering access to approved contraceptive use include; cultural disapproval of modern contraceptive methods,^7^ requirement of husband's permission for access to contraceptive services, service providers' insistence on spousal consent,^7^ and partner's disapproval.^8^, ^9^ Other causes are: misconceptions about approved modern contraceptive methods, fear of side effects such as permanent loss of fertility, and association of modern contraception with promiscuity.^1^, ^8^ These could be addressed by effective practice of drug information services in communities.^10^ Being unmarried is also associated with non‐use of modern contraceptives,^11^ which likely stems from judgmental or discriminatory attitudes of some contraceptive service providers.^8^ These major barriers to the use of approved contraceptives have consequently promoted the use of perceived alternatives which primarily include ampicillin‐cloxacillin.

## 3 EFFECTS OF MISUSE OF AMPICILLIN‐CLOXACILLIN FOR CONTRACEPTION

The redirection of ampicillin‐cloxacillin for contraception is a problem originating from inappropriate information and knowledge of contraceptive methods.^8^ The misinformation and repurposing of this antibiotic combination have increasingly become a barrier to the use of approved, effective, and modern contraceptives,^1^ with potential consequences of unplanned pregnancies and unsafe abortions.^12^ A more worrisome potential consequence of this menace is the rising incidence of microbial resistance to ampicillin‐cloxacillin in the country. Clinical strains of *Staphylococcus aureus* have shown resistance to various brands of ampicillin‐cloxacillin in Nigeria.^13^ A previous study noted 100% resistance of *Escherichia coli* to ampicillin,^14^ and another study noted high resistance of same organism to cloxacillin^14^ in Nigeria. Several other organisms have also demonstrated very high resistance to ampicillin and cloxacillin in Nigeria.^4^, ^15^ This is a threat to the effectiveness of these antibiotics in disease management.

Infections that result from resistant bacterial strains are more likely to lead to adverse outcomes, than similar infections caused by susceptible strains. These outcomes include treatment failures, increased morbidity, increased cost of care, and mortality. Other consequences are increased burden on the healthcare system, reduced quality of life, and decreased productivity of affected individuals. Antimicrobial resistance is an increasing global problem. The rising human population, along with increase in international trade and travel have potentially accelerated the spread of resistant microbial strains across the globe. Being a precursor of antimicrobial resistance, misuse of amoxicillin‐cloxacillin, which refers to its use without a clinical indication, should be discouraged through all available media. Close attention to this global threat is essential to achieving Universal Health Coverage (UHC).

## 4 IMPLICATION OF EFFECTIVE CONTRACEPTION AMONG NIGERIAN WOMEN

Use of effective contraceptives is necessary for preventing unintended and high risk pregnancies which are associated with several adverse effects on the woman, the children and the society at large. In developing countries like Nigeria, contraception is expected to play major roles in the achievement of sustainable development goals such as reduced poverty and hunger, as it potentially promotes opportunities for women's educational and economic development. Being key to population control, effective contraception is a potential means of curbing Nigeria's high population growth rate and its associated health and socio‐economic consequences. Among these consequences are: worsening of health inequalities, stretching of the limited infrastructures, and poor socio‐economic development. Reasonable measures should hence be put in place by all stakeholders to ensure appropriate and effective contraception in Nigeria.

## 5 CONCLUSION

The misperception and resultant misuse of ampicillin‐cloxacillin is a major public health threat facilitated by several barriers to modern contraceptive methods, and noncontrolled access to antibiotics. This represents a potential contribution to the rising incidence of antimicrobial resistance, and requires effective control of the drug distribution channels, and strategic behavioral interventions through targeted education.

### 5.1 Recommendations

To ensure controlled access to ampicillin ‐cloxacillin, strong political‐will and commitment is recommended for the Pharmacists' Council of Nigeria and the Federal Ministry of Health to ensure proper and strict regulation of antibiotics in the country. This will enable organized and safe drug distribution network.

Sexuality education that comprises accurate, comprehensive, and age‐appropriate information on abstinence, and a full range of approved contraceptives is key to improving knowledge and uptake of the right contraceptives. Therefore, this should be facilitated by the Federal Ministry of Women Affair, and incorporated in the teaching curriculum at all educational levels by the Federal Ministry of Education.

Provision of medically accurate public health education regarding contraceptives is a great necessity in curbing this topical problem. Integration of contraceptive awareness into health promotion services for young persons may aid in dispelling misconceptions about modern contraceptive methods, and make it more desirable for young women. Therefore, the Federal and State Ministries of Health should pay particular attention in this regard.

The Federal Ministry of Health should also ensure confidential and comprehensive contraceptive care, including access to methods of contraception for young women, irrespective of their marital status and partners' approval. Contraception education is also recommended for males to enhance their acceptance of modern methods.

Policies that discourage cultural barriers to accessing approved contraceptives by women are also highly relevant in a culture‐sensitive society like Nigeria, and should be instituted by the Ministry of Health. These policies should include nonrequirement of husband's approval for accessing contraceptive care.

## FUNDING

None declared.

## CONFLICTS OF INTEREST

The author declares no conflicts of interest.

## AUTHOR CONTRIBUTIONS

Conceptualization: Chinonyerem Ogadi Iheanacho.

Formal Analysis: Chinonyerem Ogadi Iheanacho.

Funding Acquisition: Chinonyerem Ogadi Iheanacho.

Writing ‐ Original Draft: Chinonyerem Ogadi Iheanacho.

Writing ‐ Review and Editing: Chinonyerem Ogadi Iheanacho.

## TRANSPARENCY STATEMENT

The manuscript is an honest, accurate, and transparent account of the study being reported.

## Data Availability

Data sharing is not applicable to this article as no new data were created or analyzed in this study.
